# Qualitative analysis of the impact of a lymphatic filariasis elimination programme using mass drug administration on Misima Island, Papua New Guinea

**DOI:** 10.1186/1475-2883-6-1

**Published:** 2007-01-01

**Authors:** Shona Wynd, Jaime Carron, Billy Selve, Peter A Leggat, Wayne Melrose, David N Durrheim

**Affiliations:** 1 World Health Organization Collaborating Centre for the Control of Lymphatic Filariasis, James Cook University, Douglas, Townsville, 4811, Australia; 2 United Nations Development Programmeme, Maseru, Lesotho; 3 Divine Word University, Madang, Papua New Guinea; 4 Hunter New England Population Health, Locked Bag 10, Wallsend, 2287, Australia

## Abstract

**Background:**

Papua New Guinea is the only endemic country in the Western Pacific Region that has not yet introduced a countrywide programme to eliminate lymphatic filariasis. However, on Misima Island in Milne Bay Province, government and private sectors have collaborated to implement a pilot elimination programme. Although interim evaluation indicated that the programme has been parasitologically successful, an appreciation that sustainable health gains depend on understanding and accommodating local beliefs prompted this qualitative study.

**Methods:**

We investigated Misima community members knowledge and attitudes about lymphatic filariasis and the elimination programme. A combination of focus groups and key informant interviews were used to explore participants perceptions of health; knowledge of the aetiology and symptoms of filariasis, elephantiasis and hydrocele; attitudes towards the disease and mass drug distribution; and the social structure and decision-making protocols within the villages.

**Results:**

Focus group discussions proved inferior to key informant interviews for gathering rich data. Study participants did not consider lymphatic filariasis ("*pom*") a major health problem but were generally positive about mass drug administration campaigns. A variety of conditions were frequently and incorrectly attributed to filariasis. Participants expressed the belief that individuals infected with filariasis always had visible manifestations of disease.

A common misconception was that taking drugs during campaigns provided long-term immunity against disease. The role of mosquito vectors in transmission was not generally appreciated and certain clinical presentations, particularly hydrocele, were associated with supernatural forces. Multiple adverse events were associated with mass drug administration campaigns and most study participants mentioned community members who did not participate in campaigns.

**Conclusion:**

Important issues requiring educational intervention and elimination activity modification in the Misima region were identified during this study. Research outcomes should assist Papua New Guinea in developing and implementing a national elimination strategy and inform discussions regarding the appropriateness of current elimination strategies.

## Background

Lymphatic filariasis (LF) is, after malaria, the second most common vector borne disease globally. The World Health Organization (WHO) estimated the global burden of infection to be 120 million with 1 billion people at risk of infection [1]. LF is the second most important cause of long-term disability worldwide and this provided the impetus for a global elimination programme sponsored by the WHO, non-government organizations, thedrug manufacturers Merck & Co Inc and GlaxoSmithKline, and government health departments. The programme is based upon mass community treatment with diethylcarbamazine (DEC) and Albendazole in areas where there is no co-endemicity with onchocerciasis and Ivermectin (Mectizan^®^) and Albendazole in areas where onchocerciasis and LF coexist [1].

Papua New Guinea (PNG) is the country with the greatest remaining burden of clinical and sub-clinical lymphatic filariasis (LF) in the Western Pacific Region [2]. Although the country's National Health Plan has integrated LF into its vector borne disease control programme [3], PNG is the only endemic country in the region that has not yet introduced a countrywide programme to eliminate LF. However, on Misima Island, Milne Bay Province, government and private sectors have collaborated to implement a pilot elimination programme [4]. Misima Island forms part of the Samarai Murua District of Milne Bay Province and historically this Island has experienced a high prevalence of filariasis. A survey conducted in 1997 found a prevalence rate of 56% using immunochromatographic card tests [5].

The population of Misima is relatively stable with approximately 14,000 people living in 30 widely dispersed poor rural villages. Island residents are largely dependent upon subsistence farming, which accounts for 99% of primary economic activity. At the time of this study 1% of the population was employed by the local mining industry. Formal education is limited with only 50% of children completing grade 10 and less than 2% going on to tertiary education.

Life expectancy is 57 years and this has monotonically declined since 1980 [6]. Sixty-two percent of children under 5 years of age on Misima Island are below the 20^th ^percentile of expected body weight for their age [7]. Malaria maternal health problems and respiratory disease are the major causes of morbidity and mortality [8]. Health services on the Island are limited, with 39 aid posts and 8 health centres (including the hospital in Bwagaoia) providing basic health care. Ten percent of local health facilities have closed over the past 5 years due to financial constraints [8]. Locally trained community health workers staff the aid posts but there is a reported high turnover of staff. Poor roads make access to health facilities difficult and no emergency transport service is available to remote areas.

Ward Development Committees (WDCs) consisting of 8 to 10 community leaders are the principal local management structures in villages. An elected Councillor, who serves as the principal decision-maker in community matters, chairs the WDC. However, decision-making is largely democratic, with active community involvement and debate. Additional community groups active in most communities include: Women's Fellowship groups, led by the key female community leaders, church, youth and sport groups.

Over a 5 year period the Misima Island LF elimination programme combined ongoing awareness campaigns with annual single-dose administration of diethylcarbamazine (DEC) and albendazole to all community members. This approach appeared to have impacted on overall infection rates, with interim monitoring at three separate sites in 1999 recording prevalence rates below 2% [5].

There is a growing global consensus that an understanding of local beliefs is essential if elimination programmes are to produce sustainable health benefits [9].

To date, the high levels of compliance achieved during mass drug administration (MDA) campaigns has been attributed both to the level of community engagement and to the effective utilisation of existing social structures and communication networks. Despite this belief little is actually known about the perceptions of Misima Island residents with regard to LF and the annual MDA campaigns. As part of a wider study linkingthe PNG experience with similar studies in Fiji, Myanmar and India a qualitative research study was conducted to explore Misima Island community members knowledge and perceptions of LF, obtain an understanding of community behaviour and attitudes towards the annual MDA campaigns and gather baseline data for future advocacy interventions.

## Methods

A standardised multi-centre protocol developed during a WHO workshop in 1999 was adapted specifically for this study [10]. The protocol includes a detailed question outline for use in focus group discussions and an equally detailed outline for key informant interviews. While recognising that the protocol was part of a larger study we attempted to balance concerns about the recommended methodological approach while ensuring that the study would still fulfill its obligations to the broader study.

The question outlines developed during a WHO workshop were lengthyand detailed with a fundamental focus on providing a rapid bio-medical interpretation of LF. Furthermore, the protocol recommended that onefocus group be conducted in each of the 8 villages, 4 of which wouldexclusively involve female participants and 4 of which would includeexclusively male participants. The protocol recommended that only onekey informant per village be interviewed, with an equal number ofmale and female participants overall.

Anticipating that this approach would not allow a sufficiently in-depth understanding of local perceptions of all interest groups within any given village we modified the study plan. We elected to reduce the number of participating villages to 4, while increasing the number of interviews and focus groups conducted in each of the participating villages to provide a greater depth of understanding. Four focus group discussions were undertaken in each of the villages followed by 2 to 4 key informants interviews per village in addition to translating the question outlines into the local language and establishing and including the local terminology for LF, we added a number of more general questions to the beginning of the question outlines to provide a basic understanding of local health concerns. These questions were intended to establish a rapport with participants and considered general health issues and concerns in the village.

The focus group question outline covered a number of key areas including: perceptions of health; knowledge of aetiology and symptoms of filariasis, elephantiasis, and hydrocoele; and attitudes towards the disease and mass drug distribution. The key informant question outline covered similar themes but included a more detailed additional focus on the social structure and decision-making protocols within the village. The 4 villages selected for focus group discussions and key informant interviews took account of differential remoteness, proximity to the local mining operation and health facilities available. The villages selected were: Bwagaoia, the main village on the island, in the south-east less than 1 km from the hospital; Liak, in the north of the island, approximately 2 hours by road from Bwagaoia served by a single health aid post; Bwagabwaga, in the south-west of the island and only accessible by an hour boat trip from Bwagaoia with at least an hour's walk to the nearest aid post; and Ebora, on the western tip of the island, only accessible by a 1 1/2 hour boat trip from Bwagaoia and served by a single aid post.

Each village was visited prior to commencing research activities. Meetings were held with community leaders to inform them of the reasons for the research and request permission for participation of their communities. Dates and times were organized with communities to avoid disruption of their normal activities. Four focus group discussions were then conducted in each village.

Purposive sampling was used to identify groups of teenage males, teenage females, adult males and adult females. A total of 137 volunteers participated. In addition to the focus groups, 2 to 4 key informants per village, a total of 13 individuals, were identified and interviewed. Key informants included prominent village members, such as ward councillors, pastors, Ward Development Committee members, teachers, elders and Women's Fellowship leaders.

A local health worker with extensive experience in filariasis elimination conducted the majority of interviews after receiving training in qualitative research techniques and in-depth training on the question outlines. The research assistant was trained to encourage equal participation of all participants.

Probes to clarify responses and further illuminate areas of particular importance followed key questions. Although English is widely spoken on Misima most interviews were conducted in the local Misima language to provide participants with the opportunity to communicate freely. Participants were informed of the purpose of the research and assured of the confidentiality of their individual responses.

All interviews and focus group discussions were recorded by audio cassette and later translated from Misima to English. To minimisemisunderstanding the accuracy of translation was verified by a number of English-speaking Misima Islanders. These scripts were transcribed and entered into QSR NUD*IST (N5) software for qualitative data analysis [11].

The Medical Research Advisory Committee of the PNG Department of Health and the Human Ethics Sub-Committee of James Cook University granted approval for the study.

## Results

### Identifying health issues

**Figure 1:**
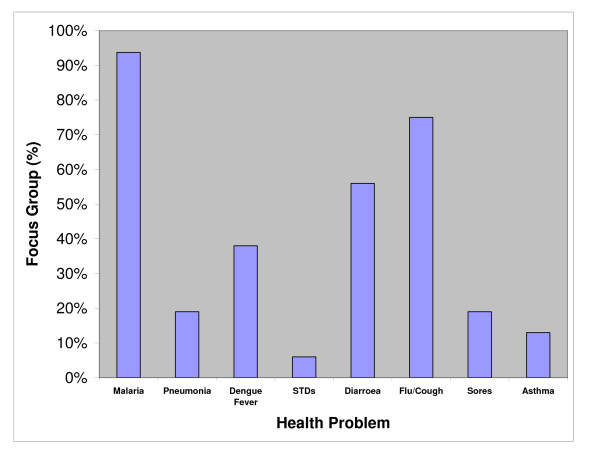
Health problems reported by village focus groups, Misima Island, PNG, Aug 2002.

When asked to provide their ranking of important local health problems none of the focus groups spontaneously identified LF. The most important local health problems reported were: malaria, flu/cough and diarrhoea (Figure 1). All but one focus group stated that malaria was their most serious health problem.

Key informants emphasized limited health infrastructure as a major problem. One councillor eloquently described lack of access to quality health services:

"The provision of good health facilities is a concern. The maintenance of the Aid Post and cleaning around the area is a problem. The Aid Post has been here 20 years and never once been maintained. There is no bed inside; we need a better supply of medicine. Transport is always a concern for emergencies."

Malaria was the specific disease most commonly mentioned by key village informants. However, poor water quality, inadequate sanitation and limited access to health facilities were major health concerns raised. A Women's Group leader stated:

"One main issue I see is a lack of proper pit toilets – there are not enough for everyone. Also (there is) a lack of clean water supply for the people in the village. We have a lot of pig waste in and around the village – some pigs are not fenced in."

Direct probing as to whether filariasis ("*pom*" – the swollen leg associated with filariasis) was a health problem consistently resulted in negative responses. When questioned about the presence of filariasis, one key informant stated:

*"I think a long, long time ago, yes. But I do not see pom nowadays"*.

One respondent recalled a death due to filariasis, while another recalled someone with a big swollen leg. One focus group participant expressed a concern that filariasis might again become a problem due to high numbers of mosquitoes, while another mentioned that some villagers were concerned about filariasis after an "educational" video was screened in their community.

### Common perceptions

Individuals commonly expressed the belief that a person infected with LF always had visible signs of the disease. When people were directly questioned about their understanding of LF, a variety of symptoms were commonly and incorrectly attributed to LF. Local names for conditions described as "filariasis" included: "*pom*" (filariasis/elephantiasis), "*balian*" (boils/abscesses), "*uliyalawa*" or "*poskila*" (cellulites), "*bwabwalata*" (oedema), and "*bwawabwawa*" (scrotal swelling).

The majority of respondents did not consider themselves at risk of LF because they had taken medication during the mass drug administration campaigns. There was a common misperception that taking the drugs provided long-term immunity. An individual in one village focus group stated that:

*"The benefit of the drug distribution is people are now in good health and they will never get sick with filariasis again in times to come"*.

Although LF was not ranked as a current health concern, respondents did not hesitate in identifying those they believed were at greater risk of contracting the disease. Generally risk was associated with not having participated in treatment campaigns, but a number of individuals in a focus group indicated that *"people who are not careful about the food they eat" *are more vulnerable.

### Source of LF

When questioned about the source of filariasis, only three focus groups identified mosquitoes as the source of illness. However, it would appear that the role of mosquitoes as a vector of any disease was poorly understood. Mosquito nets were infrequently used and mosquito breeding sites were poorly controlled, either for malaria or LF control.

Particular clinical presentations were associated with supernatural forces. In two villages chronic hydrocoele was considered to result from a curse on an individual who had stolen from a betelnut plantation. A key informant indicated that almost half of community members believed that "*sorcery and witchcraft are the cause for elephantiasis*".

### Disease treatment

In response to questions regarding treatment of LF most villagers indicated that as they no longer saw filariasis in their area they were not familiar with treatment regimes. As one key informant noted, *"For treatment...I don't know of any treatment because no-one has pom"*. Only one group mentioned that western medication was effective, while others mentioned prayer and local herbs: *"Through the prayer of God Jesus will heal these conditions by faith"; *and *"People use certain barks and roots from trees to treat such conditions"*.

### Awareness and compliance with Mass Drug Administration (MDA)

All respondents were aware of the drug distribution campaigns and indicated that the tablets were for preventing LF. Generally villagers indicated satisfaction with the drug distribution process. Local community organizations were acknowledged as being effective in delivering health programmes after local councillors granted them permission. Respondents indicated that MDAs should ideally follow local religious services when most community members were gathered together. Suggestions for improving distribution included continued involvement of village birth attendants, community based health workers, and teachers in drug distribution. One key informant stated:

*"[The best approach is] for the health department to come and consult with the health worker here, the councillor, and then the organization can be done for distribution"*.

Six of the sixteen focus groups reported that they knew of people who had refused to take medication but the majority of respondents indicated that they personally would willingly take tablets again:

*"People are happy and willing to take the pills again because they do not want to get sick with filariasis"*.

Only one focus group of young women indicated that they would not be willing to take tablets, citing medication-related adverse events and the excessive number of tablets:

*"We are not willing to take them again this year because of the sick side effects," *and *"The tablets are too many to take at one time"*.

**Figure 2:**
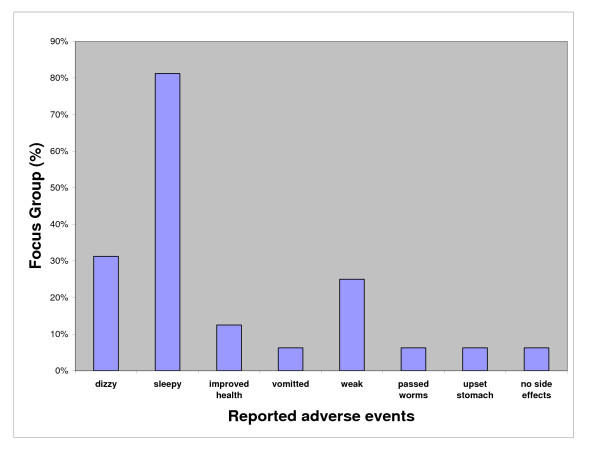
Adverse events reported as associated with mass drug administration by village focus groups, Misima Island, PNG, Aug 2002.

On probing, respondents indicated that the reasons people gave for not participating in the MDA campaign included: an excessive number of pills, vomiting as a result of the tablets, forgetfulness, or breastfeeding and pregnancy (Figure 2). Drowsiness was the most common adverse event reported, while feelings of dizziness and weakness were also reported:

"*People felt dizzy and weak. They felt like sleeping and rested after the distribution"*.

The majority of villagers indicated that affected individuals managed their own side effects by resting and sleeping. A number of "adverse events" reported actually represent benefits particularly passage of worms and altered but improved health state.

A variety of benefits were attributed to the Filariasis Elimination Programme. Eight of the sixteen focus groups indicated that they could never get sick with filariasis because the tablets had made them immune. One individual stated:

"The benefit of the drug distribution is that people are now living in good health and they believe that they will never get sick with filariasis because they have taken the pills to prevent."

Half the focus groups associated continued good health with participation in the elimination programme. A participant commented:

*"We feel that this programme is good because since this started we have had no attack of pom and we have been kept healthy"*.

Three groups mentioned that the tablets cured other sicknesses:

*"People are living in good health and this pill has cured some illness apart from pom"*.

One key informant questioned the ongoing value of the programme – given that filariasis was no longer perceived as a concern. Nevertheless he indicated his continued willingness to take the pills as a preventive measure against the disease returning:

"I myself cannot say the distribution of the filariasis tablets has done any good to us because I have never seen pom in my village. Maybe if we did not take the tablets somebody might get the sick but since we have taken the tablets this sickness will not get us. That is the good thing about the tablets."

## Discussion

Misima islanders participating in this study did not spontaneously identify lymphatic filariasis as an important current health concern. This may be due to the low prevalence of elephantiasis in the region or may reflect community definitions of disease [12]. The emphasis on rapid assessment with a qualitative survey tool focused specifically on the bio-scientific definitions of LF made it very difficult to explore the nuances of the local names, definitions and descriptions of the disease. The widespread use of the word *"pom" *to describe LF strongly indicates that local understandings of the disease are confined to outward gross manifestations of elephantiasis. In the course of this study we were left with the belief that locals had a poor understanding of micro-filarial infection and indeed the role of the mosquito as the vector.

The greater importance placed on access to adequate health facilities and life-threatening infectious diseases has also previously been identified in LF endemic areas [13]. In other settings where different understandings of filariasis exist, individuals have also associated a variety of dermatological and chronic conditions with LF [14,15]. Successful uptake of a health programme could be jeopardized where symptoms or signs incorrectly attributed to LF do not resolve, despite compliance. The importance of ongoing effective community education programmes has been noted elsewhere and is clearly indicated in the Misima context [9,16,17].

As respondents only associated filariasis with the chronic signs of LF, including hydrocoele and elephantiasis, it is not surprising that the declining prevalence of these disease manifestations was associated with perceived low personal risk. Although MDA campaigns were generally viewed positively, there were early warning signs of wanting commitment with drug-associated adverse events identified as a constraining factor. A similar trend has been detected in other countries including Thailand, India and Haiti where fever, dizziness and nausea have been associated with MDA campaigns [12,18,19].

The limited appreciation of the role of vectors found is not unusual in areas where elimination has relied on MDA campaigns [20,14,21]. Certain "adverse events" associated with treatment administration, particularly "passing worms", could serve as a motivating factor if community members were informed that this was an additional therapeutic benefit.

While traditional hierarchical community structures persist with power for decision making residing with a limited number of traditional leaders, drug distribution programmes may remain successful despite different understandings of LF in the affected communities. To date, the Misima campaign has relied very heavily on the assumption that by working through local traditional decision making structures, compliance will remain high. However, to achieve sustainable improvements in health, efforts to bridge the gap between local health knowledge systems and western biomedical models will become increasingly important. The importance ascribed to supernatural influences and limited understanding of the biomedical model of disease transmission appears to influence participation in disease control efforts and treatments both in PNG and in other contexts [9,17]. While our capacity to comment in-depth on the Misima context is constrained by the limited insights provided by the recommended methodology, we did identify what could be an early departure from more traditional hierarchical decision-making processes. One focus group, of young women in particular, made a number of comments indicating that personal considerations should enjoy preference to the directives of local traditional authorities.

As has been discussed earlier, the quality and depth of information gathered using the question outlines developed as part of a standardised multi-centre protocol. Important limitations included language barriers, limited ability of the instrument to provide in-depth understandings of cultural norms and practices, and short time prescribed. Focus group discussions were constrained by the need for an interpreter. The cumbersome process of translation and back translation, focus group participants apparent concern about providing incorrect responses despite reassurances that all responses were valid and an observed preference in all focus groups to establish consensus before providing a group response, influenced data-collection. These limitations did not permit a thorough exploration of local understandings of LF, its origins or relevant community behaviour. A thorough understanding of all of these elements is essential for the development of appropriate educational materials which, in addition to achieving longer term sustainability, foster a balance between western and local health knowledge systems.

Many of the questions included in the standardised multi-centre protocol were developed for populations where chronic manifestations of LF are still common, which is not the case on Misima Island. The large number of prompts relating specifically to elephantiasis and hydrocoele leave limited time to probe more subtle presentations and respondents often indicated that these questions were irrelevant. While interview questions presupposed a shared definition of LF, respondents had difficulty relating to the bio-medical interpretation of the disease that underpinned the question outline.

Furthermore, because of the low prevalence of the disease in the region, individuals struggled to remember the details of their past experiences, if any, with the disease. Some modification of the assessment instrument in accordance with local disease epidemiology and understandings is essential. Despite these constraints, our experience suggests that richer data resulted from in-depth interviews with key informants than focus group discussions.

## Conclusion

This investigation identified knowledge gaps requiring educational intervention, perceptions and community behaviours that may require modifications in LF elimination activity in the Misima region. These findings will be of value in Papua New Guinea as the national LF elimination strategy is developed and implemented. Although compliance and community satisfaction with the LF elimination programme in Misima appeared high, there was a need for an effective education programme focusing on LF transmission and prevention. The central necessity of community involvement in developing educational strategies that reflect local understandings and interpretations of the disease should not be overlooked.

A particular concern was that Misima islanders associated LF infection only with the chronic visible manifestation of filariasis and other unrelated diseases. Another potentially detrimental belief is that tablet consumption provides lifelong immunity against contracting infection and this may contribute to the neglect of personal vector control measures. Sustainable LF treatment and prevention interventions require a broad understanding of local disease perceptions, including mode of transmission, consequences of infection and means of prevention.

## Competing interests

The author(s) declare that they have no competing interests.

## Authors' contributions

SW, PL, WM, DD conceived the study and developed the study design. Fieldwork and data collection was conducted by JC, BS. Principal dataanalysis was performed by SW and JC. All authors contributed to interpretation of results and manuscript preparation.
